# Is the “end‐of‐study guess” a valid measure of sham blinding during transcranial direct current stimulation?

**DOI:** 10.1111/ejn.15018

**Published:** 2020-11-20

**Authors:** Christopher Turner, Catherine Jackson, Gemma Learmonth

**Affiliations:** ^1^ School of Psychology University of Glasgow Glasgow UK; ^2^ Centre for Cognitive Neuroimaging Institute of Neuroscience and Psychology University of Glasgow Glasgow UK

**Keywords:** placebo, primary motor cortex, reaction time, sham, tDCS

## Abstract

Studies using transcranial direct current stimulation (tDCS) typically incorporate a *fade‐in*, *short‐stimulation*, *fade‐out* sham (placebo) protocol, which is assumed to be indistinct from a 10–30 min active protocol on the scalp. However, many studies report that participants can dissociate active stimulation from sham, even during low‐intensity 1 mA currents. We recently identified differences in the perception of an active (10 min of 1 mA) and a sham (20 s of 1 mA) protocol that lasted for 5 min after the cessation of sham. In the present study we assessed whether delivery of a higher‐intensity 2 mA current would exacerbate these differences. Two protocols were delivered to 32 adults in a double‐blinded, within‐subjects design (*active:* 10 min of 2 mA, and *sham:* 20 s of 2 mA), with the anode over the left primary motor cortex and the cathode on the right forehead. Participants were asked “*Is the stimulation on*?” and “*How sure are you?*” at 30 s intervals during and after stimulation. The differences between active and sham were more consistent and sustained during 2 mA than during 1 mA. We then quantified how well participants were able to track the presence and absence of stimulation (i.e. their sensitivity) during the experiment using cross‐correlations. Current strength was a good classifier of sensitivity during active tDCS, but exhibited only moderate specificity during sham. The accuracy of the *end‐of‐study guess* was no better than chance at predicting sensitivity. Our results indicate that the traditional end‐of‐study guess poorly reflects the sensitivity of participants to stimulation, and may not be a valid method of assessing sham blinding.

## Graphical abstract

We quantified how well participants were able to track the presence and absence of a tDCS current (i.e. their sensitivity), using cross‐correlations. Current strength was a good classifier of sensitivity (higher for 2mA than 1mA), but the accuracy of their *end‐of‐study guess* was no better than chance at predicting sensitivity. The traditional end‐of‐study guess method poorly reflects the sensitivity of participants to stimulation, and may not be a valid method of assessing sham blinding.

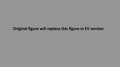

**Abbreviations**
AUC: area under the curve
CI: confidence interval
DC: direct current
ROC: receiver operating characteristic
RT: reaction time
*SD*: standard deviation
tDCS: transcranial direct current stimulation

## 1 INTRODUCTION

Transcranial direct current stimulation (tDCS) is a popular method of neuromodulation that involves the application of weak electric currents to the scalp. These currents are thought to induce temporary changes in the excitability of the underlying cortex, and can subsequently effect changes in behaviours that are regulated by these cortical areas. The majority of clinical and non‐clinical studies involving tDCS incorporate some form of placebo control condition into their experimental designs, e.g. the “*fade‐in, short‐simulation, fade‐out*” sham protocol (Fonteneau et al., 2019; Nitsche et al., 2003). This type of sham protocol is claimed to be indistinguishable from longer, active periods of stimulation (e.g. 10–30 min) on the scalp, and as such, participants are assumed to be blind to the condition that is being administered (Ambrus et al., 2012; Gandiga et al., 2006; Palm et al., 2013; Poreisz et al., 2007; Russo et al., 2013; Tang et al., 2016).

However, a growing number of studies have reported a failure of sham blinding during both high‐ (2 mA; O’Connell et al., 2012; Wallace et al., 2016) and lower‐intensity (1–1.5 mA) tDCS (Benwell et al., 2015; Goldman et al., 2011; Greinacher et al., 2019; Kessler et al., 2012; Turi et al., 2019). One potential source of these mixed results could stem from the method that is used to assess the success of sham blinding across studies. This measure typically takes the form of a post‐stimulation questionnaire to (a) directly probe if participants can identify whether they had received active or sham tDCS (the “*end‐of‐study‐guess*” test) and (b) to quantify the strength of sensations (e.g. tingling or burning) or other side‐effects (e.g. changes in mood or concentration levels) experienced during stimulation.

It is assumed that these questionnaires represent a valid measure of sham blinding (Antal et al., 2017), yet the responses that are obtained may depend on a range of factors. For example, in the case of multi‐session experiments, participants may find it difficult to recall their experiences in prior sessions, particularly when the sessions were many days or weeks apart. They may have taken part in previous electrical stimulation experiments and may know what sensations to expect (Ambrus et al., 2012). They may be students who have learned that active protocols tend to be compared to a sham condition, or have gained insight from the experimenter (perhaps disclosure of this information was mandated by an ethics committee) that multiple and different stimulation protocols would be administered. Finally, the questionnaires may not sufficiently clarify technical jargon such as “sham” or “placebo” in lay terms, or may fail to specify whether participants are expected to report only the side‐effects that were experienced for a prolonged period of time, or those felt only briefly. In short, the end‐of‐study questionnaires represent a single snapshot in time—by definition, after the experiment has ended—and yet few studies ask participants to explain the reasons for their choice.

We recently showed in Greinacher et al. (2019) that sham blinding was compromised during 1 mA tDCS using a novel method of assessment. Participants undertook a simple, forced‐choice reaction time task during 10 min of 1 mA active tDCS and a 20 s 1 mA sham protocol, based on Minarik et al. (2016). None of the participants had received electrical stimulation before, and the two protocols were carefully double‐blinded during their application. At regular, 30 s intervals during the 16 min experiment participants were asked “*Is the stimulation on?*” (yes/no) and *“How sure are you?”* (0–10 scale). We identified a prolonged period of difference in the perception of active and sham protocols at a group level. Participants were confident that stimulation during the sham protocol switched off after 2 mins, while they remained confident that stimulation during the active protocol was still switched on until around 11.5 mins after onset. We argue that probing the success of sham blinding online, during the course of the tDCS delivery, enables researchers to identify differences in perception that might influence the performance of the task that is being undertaken at that point. For example, participants may reduce their effort on the task when they sense the stimulation has ended, leading to differences in performance during the active and sham conditions as a result of failed sham blinding, rather than induced neural effects of tDCS.

Our results in Greinacher et al. (2019) partially conflict with those observed in Ambrus et al. (2012), where multiple (although fewer, at only 7) online probes were presented during stimulation. Participants were asked to report the location and strength of scalp sensations every 1.75 min during 10 min of 1 mA anodal and cathodal tDCS, plus a 30 s sham condition. Overall, in line with our results, strength ratings gradually reduced over time, and this reduction was faster in the sham condition compared to anodal and cathodal (the drop was significant by 2.25 min in sham, 4 min in anodal and 5.75 min in cathodal). However, after excluding the 12 tDCS *investigators* who had participated as subjects, there were no differences in strength ratings between the three stimulation protocols in either the naïve or experienced participant groups. These participants were also unable to tell whether they had received real or placebo stimulation. Although our participants were similarly naive to tDCS, we found clear differences in the perception of active anodal and sham stimulation at this low‐intensity current strength. However, it should be noted that different questions were used in the two studies which may have contributed to this difference: an analogue scale from *no sensation* to *extreme discomfort* in Ambrus et al. (2012), and a binary choice of whether the stimulation was switched on, plus a confidence rating, in Greinacher et al. (2019).

Ensuring that participants are blind to the protocols that are administered is an essential facet of tDCS experimental design. If participants are able to identify the condition, or the period of stimulation within each condition, during which they are receiving active stimulation, they may alter their behaviour in line with the expected outcome. For example, they may speed up during a reaction time task or expend more effort in memorising sequences when they feel the stimulation on their scalp. Alternatively, if they can feel the stimulation in one protocol more than another and are distracted from the task, the outcome measures of interest may be contaminated by sensation‐related artifact. This could result in researchers mistaking such behavioural changes for induced neural effects of tDCS and, particularly within clinical practice, may lead to tDCS being unjustifiably recommended as a therapeutic intervention for clinical conditions.

In this study, we aimed to extend our results in Greinacher et al. (2019) by repeating the experiment at a higher‐intensity current of 2 mA rather than 1 mA. We predicted that participants would find it easier to identify the presence and absence of stimulation during both active and sham tDCS when 2 mA is delivered, due to stronger sensations on the scalp. Specifically, participants were expected to report that the stimulation was switched on for a longer duration in the active condition compared to our observations during 1 mA. Finally, although in Greinacher et al. (2019) we found no tDCS‐induced changes in reaction times during the forced‐choice behavioural task, we hypothesised that a higher, 2 mA current may lead to a reduction of reaction times during this task.

## 2 METHODS

### 2.1 Pre‐registration

The pre‐registered study protocol, information sheet, questionnaires and full datasets are available at https://osf.io/4ath9/.

### 2.2 Participants

Thirty‐two participants were tested (mean age = 25.69 years, range = 20–41, 22 females). All participants were right‐handed with normal or corrected‐to‐normal vision, had no contraindications to tDCS (Rossi et al., 2009), and had not taken part in an electrical stimulation study before. The sample size was based on a planned one‐tailed, repeated measures *t* test on the reaction time data, where an effect size of *d* = 0.45 was expected (as observed in Minarik et al., 2016), power = 0.8 and *α* = 0.05. The study was approved by the University of Glasgow College of Science and Engineering ethics committee and performed in accordance with the Declaration of Helsinki. All participants provided written informed consent.

### 2.3 Transcranial direct current stimulation

**FIGURE 1:**
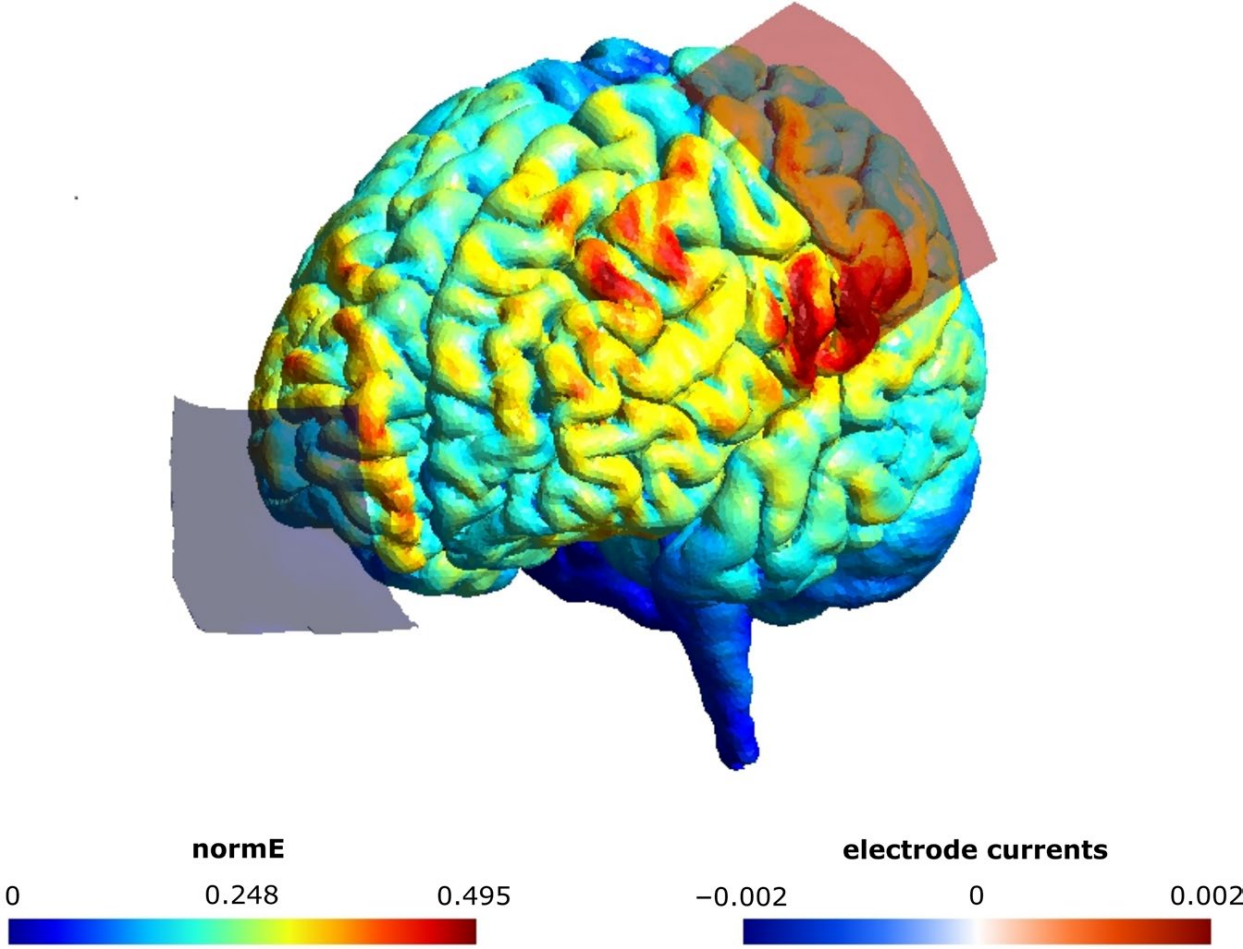
Electrode montage and simulated current flow (SimNIBS 3.1.0, Thielscher et al., 2015). A 5 × 7 cm anode was centred vertically on the left primary motor cortex (C3) and a 5 × 7 cm cathode was positioned horizontally over the right forehead. The norm of the electric field strength (normE) is shown in V/m and the current induced by each electrode in mA

A direct current was applied using a battery‐driven constant current stimulator (NeuroConn GmbH). The parameters were identical to those in Greinacher et al. (2019), although a 2 mA current was delivered here rather than 1 mA. Two tDCS protocols (*active* and *sham*) were applied in a double‐blinded, counterbalanced, within‐subjects design. Double blinding was achieved using the study mode of the NeuroConn stimulator. Pre‐allocated 5‐digit codes were entered into the device, which initiated either the active or sham protocol. The active protocol involved 10 min of 2 mA stimulation and sham 20 s of 2 mA stimulation, with both protocols including an additional 30 s ramp‐up and 30 s ramp‐down period. In both protocols, the anode was centred over the left primary motor cortex (C3) and the cathode placed horizontally over the right forehead (Figure 1). Both carbon rubber electrodes measured 5 × 7 cm, were placed inside 0.9% NaCl saline soaked sponges and were attached to the scalp using a total of four rubber bands (two around the forehead and pinned at the back of the head to hold the cathode in place, and two around the chin and pinned at the top of the head to hold the anode in place). Both bands lay adjacent to one another to ensure full contact between the electrode and the scalp. The amount of saline added to the sponges was not standardised. Sponges were fully immersed in saline and then manually squeezed until there was no excess saline dripping from them prior to placing them on the scalp. The mean impedance at the start of stimulation was 4.27 kΩ (range = 2.2–9.6 kΩ).

### 2.4 Behavioural task

Based on Minarik et al. (2016), and identical to Greinacher et al. (2019), participants completed a simple, forced‐choice reaction time task before, during and after stimulation. Stimulus materials are available at https://osf.io/2zwhg/. In Block 1 (baseline) there were 100 trials (lasting around 3.5 min), where either a diamond or square appeared in the centre of the screen for 100 ms, followed by a fixation cross of variable duration (1,700–2,100 ms). Participants were instructed to respond as fast as possible using their right hand, by clicking the left mouse button to respond to a diamond and the right button for a square. In Blocks 2–4 two probe questions were asked after every 30 s, with each question being presented for a fixed duration of 4,500 ms. For the first question (“*Is the stimulation on?”*) participants were instructed to click within either the *yes* or *no* box on the screen. For the second question (“*How sure are you?*”) participants were instructed to click along a visual analogue scale ranging from 0 to 10, where 0 = very unsure and 10 = very sure. There were 32 probe points in total during Blocks 2–4 spanning a total of 16 min.

### 2.5 Procedure

**FIGURE 2:**
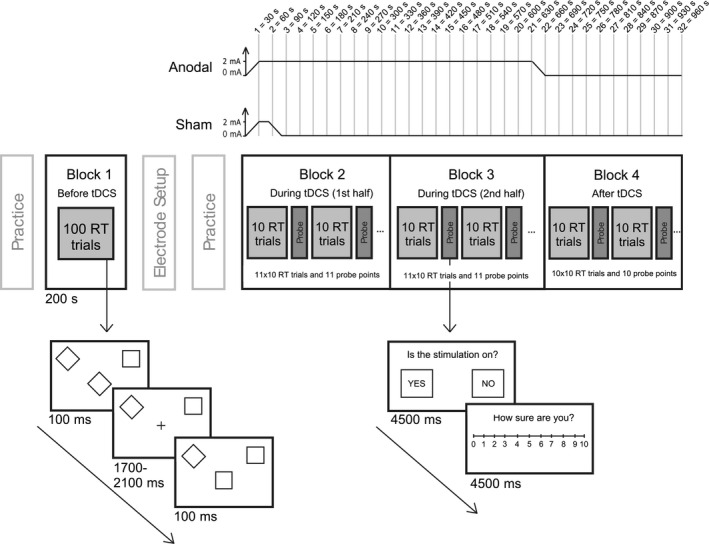
Illustration of the experimental design

Thirty practice trials were completed, followed by the set of Block 1 trials (see Figure 2). The electrodes were then positioned and fixed on the scalp. The resistance was checked and lowered if found to be >10 kΩ by (a) checking that the hair was fully parted under the anode, (b) tightening the rubber bands by adjusting the pins and (c) in only a few cases adding extra saline to the electrodes using a syringe. At the start of Block 2, the 30 s tDCS ramp up was initiated at exactly the same time as the behavioural task was started. Blocks 2–4 were then completed continuously, without breaks, and the end of Block 3 coincided with the end of the 30 s ramp‐down period in the active condition. Block 4 was completed entirely post‐stimulation in both protocols. For each participant, the two sessions were performed at least 24 hr apart. At the end of each session, participants completed a questionnaire which probed their ratings of headache, tingling, itching, burning and pain during stimulation on a scale of 1 = not at all to 5 = very strongly. Finally, they were also asked to guess which of the two sessions had involved sham at the end of the experiment.

### 2.6 Analyses

#### 2.6.1 Reaction times

The median reaction time for correct trials was calculated separately for blocks 1–4. The baseline (Block 1) median RT was then subtracted from each subsequent block to create ΔRT values for blocks 2–4. As per the pre‐registered analysis plan, a series of three repeated measures *t* tests were performed to compare the ΔRT between the active and sham stimulation conditions in blocks 2, 3 and 4. It was expected that a larger ΔRT would be elicited by the anodal condition compared to the sham condition in all experimental blocks, and therefore one‐tailed tests were used. The ΔRT was then compared between the present 2 mA dataset and the 1 mA dataset from Greinacher et al., (2019), separately for blocks 2–4, using 3× one‐tailed independent samples *t* tests.

#### 2.6.2 Effectiveness of sham blinding

**FIGURE 3:**
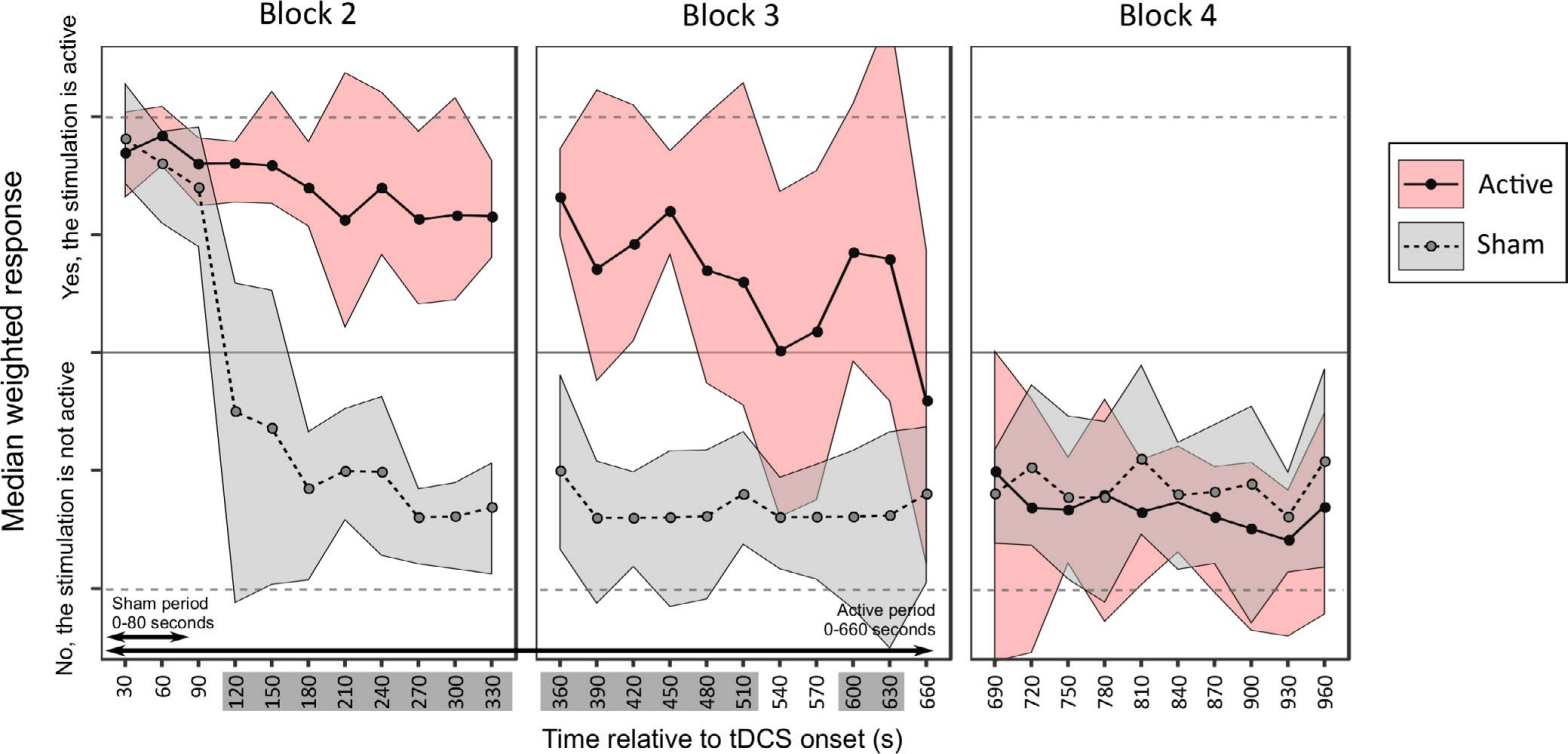
Median weighted scores with 95% confidence intervals for the active (pink) and sham (grey) protocols. The time points with non‐overlapping confidence intervals between the two protocols are highlighted in dark grey on the*x*‐axis

A weighted score was created for each probe point, per participant, where a “yes” response to the question “*Is the stimulation on?*” was assigned a value of +1, and a “no” response a value of −1. This was multiplied by the confidence rating provided in response to the question “*How sure are you?*”, and the weighted scores therefore ranged from +10 = high confidence that the stimulation is on, to −10 = high confidence that it is off. 95% confidence intervals were then bootstrapped for each probe point using 5,000 permutations of the data, separately for active and sham (Figure 3). Wilcoxon signed‐rank tests were performed to compare the strength ratings of the 5 sensory side‐effects across the two tDCS protocols.

#### 2.6.3 Cross‐correlations

**FIGURE 4:**
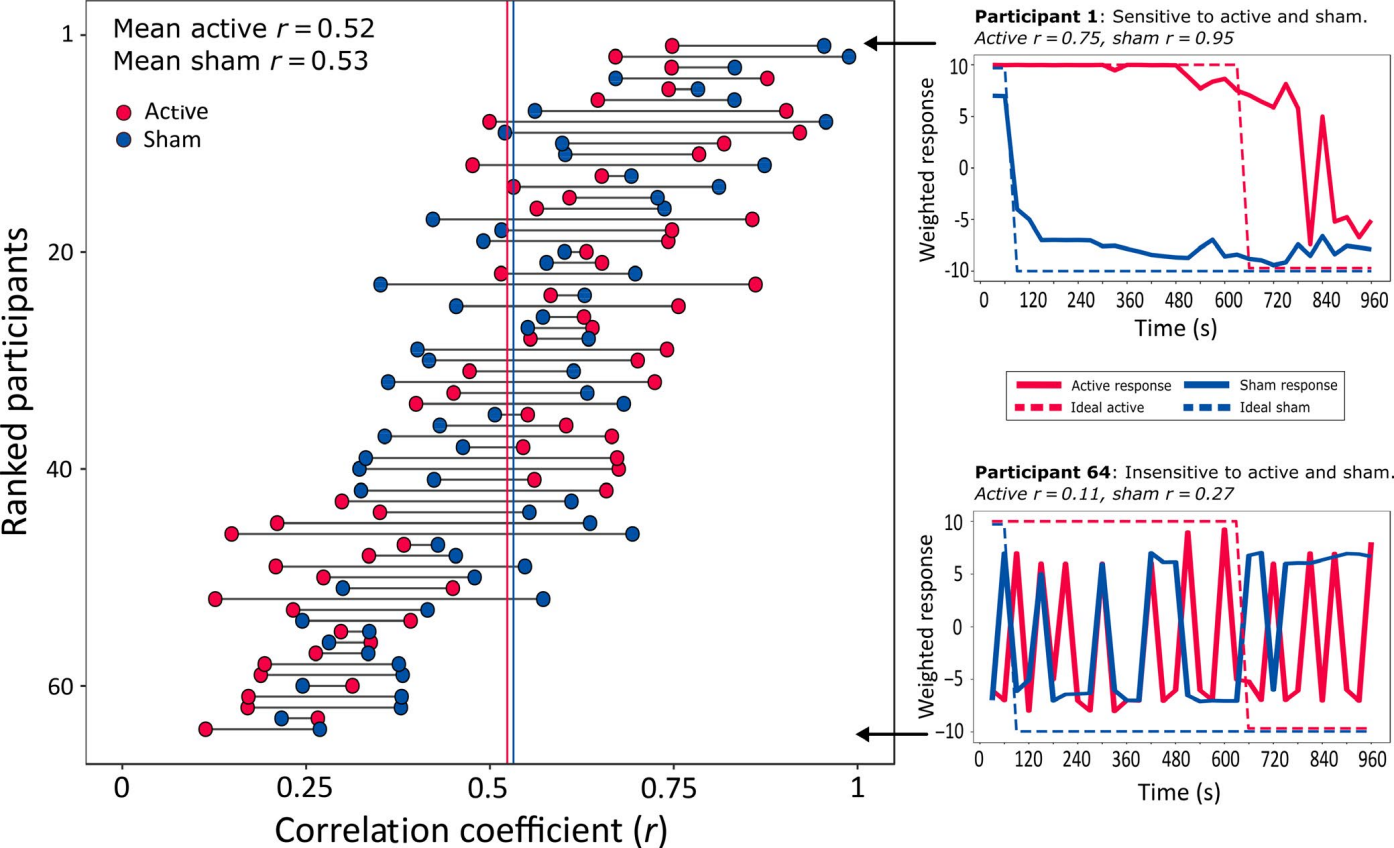
Peak cross‐correlation coefficients. The left panel shows all 64 participants ranked by the sum of their Pearson's correlation coefficients for the active and sham protocols. Mean*r*‐values for active and sham are shown as red and blue vertical lines respectively. The panels on the right show the response curves from two participants to illustrate the range of sensitivities that were observed across individuals. The highest ranked participant (top right) was highly sensitive to the onset and offset of stimulation during both active and sham protocols. The participant with the lowest sum of coefficients (lower right) responded randomly during both protocols and was thus considered insensitive to the presence of active and sham

Cross‐correlations were used to quantify the similarity between two time‐series datasets in the form of a correlation coefficient. Each participant's response curve was cross‐correlated (separately for active and sham), with the “ideal response” curve for that condition. The ideal response represents the curve that we would expect to observe if the participant was able to report the presence and absence of stimulation with 100% accuracy (see examples of this in Figure 4, right panels). To test the specificity of this analysis method we also performed the cross‐correlations with the ideal response for the *opposite* condition (i.e. the response curves for the *active* condition compared to the ideal *sham*, and vice versa). This resulted in a *congruent* and *incongruent* cross‐correlation coefficient for each participant during active and sham stimulation, where *r* = 1 represents a perfect positive correlation between the participant's response and the ideal response.

The cross‐correlations were performed by first detrending the data vectors then using the *xcorr* function in Matlab with the “*scaleopt”* parameter set to “*coeff*.” A sliding window was applied to the whole 15 min time frame (from −32 to 32 lags) because we anticipated that there may be a delay between the stimulation switching on/off and the participants reporting this change. The “ideal” curve was slid from −32 to +32 lags relative to the participants’ response curve and Pearson's correlation coefficients were extracted for each lag. The maximum (peak) coefficient was extracted and used in subsequent analyses (n.b. peak correlations were extracted at a median lag of +1 for the active protocol and −1 for sham). The cross‐correlation analysis was performed on both the present dataset obtained during 2 mA tDCS and the 1 mA dataset from Greinacher et al. (2019).

#### 2.6.4 Specificity analysis (ROC curves)

Finally, in addition to indexing the sensitivity of participants to the presence of stimulation, we also assessed the specificity of this analysis method. Receiver operating characteristic (ROC) curves were used to assess the specificity of the three binary‐outcome classifiers described above: a classification of the correlation coefficients as belonging to (a) the congruent or incongruent correlation analysis, (b) the 1 or 2 mA dataset, or (c) the participants who were correct or incorrect in their end‐of‐study guess. The area under the curve (AUC) provides a measure of how well a model can distinguish between two states, where a value of 1.0 represents a perfect classifier and 0.5 represents random chance.

## 3 RESULTS

### 3.1 Reaction times

There were no ΔRT differences between active and sham tDCS in any of the three blocks (Block 2: *t*(31) = −0.04, *p* = .49, *d* = 0.007; Block 3: *t*(31) = 0.46, *p* = .33, *d* = 0.08; Block 4: *t*(31) = −0.18, *p* = .43, *d* = 0.03, adjusted alpha = 0.017, see Figure S1). No differences in ΔRT were observed when comparing the present 2 mA dataset and the 1 mA data from Greinacher et al. (2019) in either the active or sham protocol (minimum *p*‐value = 0.28, maximum *d* = 0.28, adjusted alpha = 0.017).

### 3.2 Effectiveness of sham‐blinding during 2 mA tDCS

The confidence intervals for active and sham overlapped for the first 90 s, where participants reported with high confidence that the stimulation was switched on in both protocols. By 2 min after onset, participants were confident that the sham stimulation had ended, whereas they reported maintained confidence that the active tDCS was still switched on until 510 s (8.5 min) after onset and again from 600 to 630 s (10–10.5 min) post‐onset. By the end of Block 3, at 660 s (11 min after onset), coinciding with the point at which the active tDCS had fully ramped down, participants reported that the stimulation had ended in both conditions. This was an increase of three non‐overlapping time points compared with the 1 mA dataset in Greinacher et al. (2019), although the confidence intervals for the two studies overlapped during both active and sham conditions, mainly due to inter‐subject variability.

Similar to Greinacher et al. (2019), itching was stronger in the active condition relative to sham (*Z* = −3.13, *p* = .002) but there were no differences for headache, tingling, burning, or pain (minimum *p* = .197).

### 3.3 Cross‐correlation sensitivity analysis

Although the results highlighted clear group‐level differences in the time‐course of perceptions associated with active and sham tDCS, we noted a high degree of inter‐individual variability in the sensitivity of participants to the presence or absence of stimulation. As an exploratory follow‐up, we formally assessed this variability using cross‐correlations.

Figure 4 (left panel) shows the peak correlation coefficients of each of the 64 individual participants tested. The mean peak coefficient was moderately large for both the active (*r* = .52, *SD* = 0.22) and sham conditions (*r* = .53, *SD* = 0.19), with no difference observed between tDCS protocols (paired samples *t* test, active vs. sham: *t*(63) = 0.27, *p* = .79). However, there was high variability across participants. The panels on the right of Figure 4 show the data from two individual participants: one with high sensitivity to both active and sham (top right panel; *r*_active_ = .75, *r*_sham_ = .95), and another individual with low sensitivity to both stimulation conditions (lower right panel; *r*_active_ = .11, *r*_sham_ = .27). There was a small within‐participant correlation of sensitivity during the active and sham stimulation conditions *r*(64) = .26, *p* = .039.

### 3.4 Higher sensitivity to 2 mA than 1 mA tDCS

**FIGURE 5:**
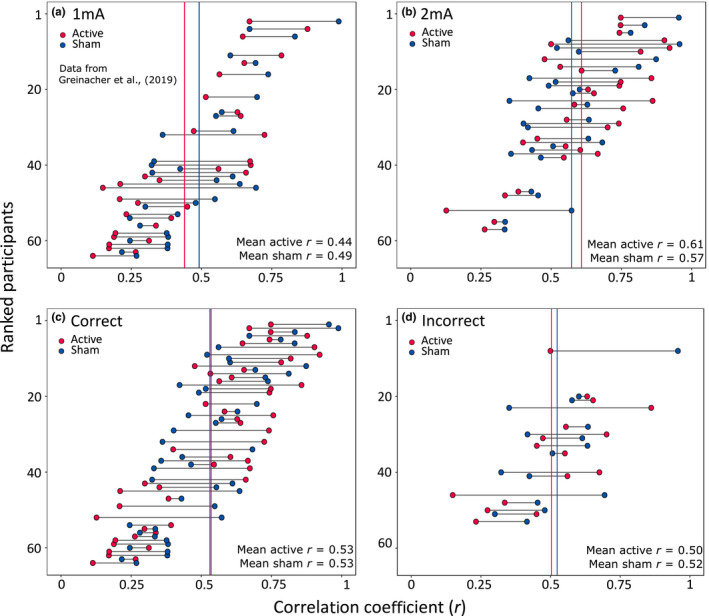
Panels (a) and (b): Peak cross correlations separated by current strength (a = 1 mA, b = 2 mA). Panels c and d: Peak cross correlations separated by the accuracy of the end‐of‐study guess regarding which of the two sessions had involved sham (c = correct participants, d = incorrect participants)

The effect of current strength on sensitivity was then assessed (Figure 5, panels a & b). We predicted that sensitivity (i.e. the correlation between the participants’ response and the congruent ideal response) would be higher during 2 mA compared to 1 mA stimulation due to stronger sensations on the scalp. As expected, a mixed ANOVA (2× current strengths and 2× stimulation types) identified a large main effect of current strength *F*(1,62) = 10.62, *p* = .002, *η*_p_^2^ = 0.146. There was no effect of stimulation type *F*(1,62) = 0.07, *p* = .79, *η*_p_^2^ = 0.001 and no interaction between current strength and stimulation type *F*(1,62) = 1.95, *p* = .17, *η*_p_^2^ = 0.03.

### 3.5 No relationship between sensitivity and end‐of study guess

We reasoned that the cross‐correlation measure of sensitivity should also correspond with the binary response provided at the end of the study, in which participants were asked to guess which of the two sessions had involved sham stimulation (Figure 5, panels c & d). Specifically, participants who correctly identified the sham session were predicted to be able to track the presence and absence of stimulation more closely in both tDCS protocols than those who were incorrect. In total, 75% (48/64) of our participants responded correctly, with similar results during 1 mA (25/32 = 78.1%) and 2 mA tDCS (23/32 = 71.9%). Of note, there was no overall difference in the sensitivity of participants who guessed correctly compared to those who were incorrect during active stimulation: Welch's *t* test *t*(32.1) = 0.47, *p* = .64 or during sham: *t*(29.9) = 0.22, *p* = .83. The individuals who guessed correctly had a wide range of sensitivities, some with very high correlation coefficients (max *r* = .99) and others with low coefficients (min *r* = .11, Figure 5c). Although none of the 16 individuals who were *incorrect* in their end‐of‐study guess were consistently highly sensitive to both protocols, they did not cluster around the lower correlation coefficients like we had expected (max *r* = .96, min *r* = .15, Figure 5d).

### 3.6 Relationship between sensitvity and reaction times

We investigated whether the participants’ sensitivity might be related to the change in reaction time at the end of the 10 min stimulation period (block 3). Four Pearson's correlations were performed, for 1 mA active, 2 mA active, 1 mA sham, and 2 mA sham. We found a moderately strong positive Pearson's correlation of *r* = .62 *p* < .001 during 2 mA active stimulation, where the highly sensitive participants tend to be those who *slowed* in their reaction times during a prolonged period of high‐intensity stimulation. No correlations were identified during the shorter high‐intensity stimulation protocol: 2 mA sham (*r* = −.07, *p* = .72), nor the two lower‐intensity tDCS conditions: 1 mA active (*r* = −0.29, *p* = .11) or 1 mA sham (*r* = .04, *p* = .81).

### 3.7 Specificity analysis

A 2 × 2 ANOVA (two stimulation types and two congruence types) identified a main effect of correlation congruence (*F*(1,63) = 55.87, *p* < .001, *η*_p_^2^ = 0.47), indicating that response curves were specific to the stimulation protocol that the participant was receiving (mean *congruent*: *r* = .53, 95% CI = [0.49, 0.57] and *incongruent: r* = .39, 95% CI = [0.37, 0.41]). There was no main effect of stimulation type (*F*(1,63) = 2.14, *p* = .15, *η*_p_^2^ = 0.33) and no stimulation × congruence interaction (*F*(1,63) = 0.28, *p* = .6, *η*_p_^2^ = 0.004).

#### 3.7.1 Cross‐correlation congruence

**FIGURE 6:**
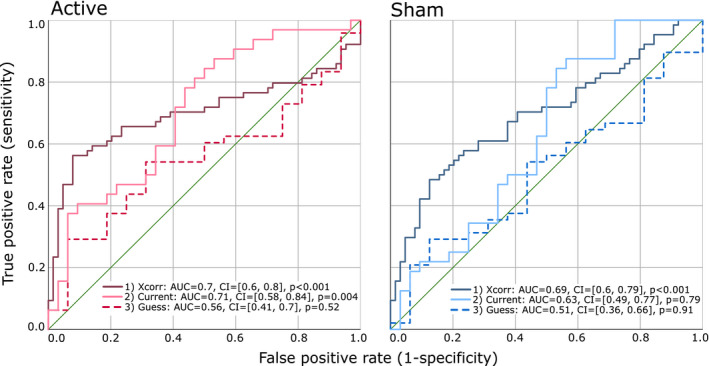
ROC curves demonstrating the sensitivity and specificity of three classification methods for the active and sham tDCS protocols: (1) cross‐correlations, (2) current strength and (3) the end‐of‐study guess

The ROC curve (Figure 6) indicates that congruence is a good predictor of correlation strength (sensitivity), with a classification accuracy of around 70% (active: AUC = 0.7, 95% CI = [0.6, 0.8], *p* < .001; sham: AUC = 0.69, 95% CI = [0.6, 0.79], *p* < .001).

#### 3.7.2 Current strength

Current strength is a good predictor of sensitivity, but only during active stimulation, with an accuracy of 71% (AUC = 0.71, 95% CI = [0.58, 0.84], *p* = .004). Current strength is a less effective predictor of sensitivity during sham stimulation (AUC = 0.63, 95% CI = [0.49, 0.77], *p* = .079).

#### 3.7.3 End‐of‐study guess

The accuracy of the end‐of‐study guess failed to predict sensitivity in either stimulation condition, with only random‐chance levels of accuracy (active: AUC = 0.56, 95% CI = [0.41, 0.7], *p* = .52; sham: AUC = 0.51, 95% CI = [0.36, 0.66], *p* = .91).

## 4 DISCUSSION

We have demonstrated that participants are able to identify that a 10 min 2 mA tDCS current is being delivered for a longer period of time compared to a 20 s 2 mA sham protocol. As predicted, the perceived differences between active and sham tDCS were more pronounced during high‐intensity 2 mA stimulation compared to the 1 mA current that was applied in Greinacher et al. (2019), with an additional three time points with non‐overlapping confidence intervals in the second half of the 10 min stimulation period during 2 mA tDCS. Using a novel method of quantifying the sensitivity of participants using cross‐correlations, we found that current strength was a good classifier of sensitivity during active tDCS, but was only a moderately accurate classifier during sham. Finally, the accuracy of the binary end‐of‐study guess was no better than chance at classifying sensitivity during either active or sham stimulation, raising important questions regarding the validity of this method of assessing sham‐blinding.

Also aligned with our previous findings using 1 mA tDCS in Greinacher et al. (2019), we found no beneficial effects of 10 min of 2 mA anodal stimulation over the left primary motor cortex in improving reaction times. This adds to a growing number of studies that have failed to show facilitatory effects of tDCS to the primary motor cortex (Apšvalka et al., 2018; Conley et al., 2015, 2016; Greinacher et al., 2019; Horvath et al., 2016; Turkakin et al., 2018; Wrightson et al., 2020).

We were able to uniquely quantify the sensitivity of participants to the presence of active and sham stimulation throughout the course of the experiment by using cross‐correlations. Participants with large correlation coefficients were considered to be sensitive in their ability to track the presence (and absence) of stimulation, and those with small coefficients as insensitive. This method adds key information regarding the time‐course of sham blinding which is unavailable to researchers using only the standard methods of assessing placebo control, namely administering questionnaires at the end of the study. These post‐study questionnaires usually incorporate a binary probe question to assess whether participants can identify if an active or sham protocol was delivered, or which of multiple sessions had involved active or sham in the case of repeated measures designs. Since our cross‐correlation measure quantified how well participants could track the presence of stimulation during both 10 min of active anodal and a 20 s sham, we expected to observe an association between this measure and the accuracy of their end‐of study guess. Specifically, that a high sensitivity would lead to an increased likelihood of correctly dissociating the two conditions. On the contrary, our results showed no association between the end‐of‐study guess accuracy and sensitivity to either active or sham tDCS, and the end‐of‐study guess was, in fact, a poor classifier of sensitivity.

The end‐of‐study questionnaire method of assessing sham blinding is undoubtedly the most popular method used in electrical brain stimulation studies (Antal et al., 2017). However if, as we show here, the end‐of‐study guess does not reflect the participants’ sensitivity to the presence of stimulation, what might this guess represent? There are many potential explanations for this discrepancy, such as the presence of confusing and technical terminology in the questionnaires (e.g. did participants understand the distinction between “sham” and “active”?, and did they know whether to report fluctuating and/or sustained scalp sensations?, perhaps they had a poor memory of the prior sessions, and were influenced by prior experience or knowledge of tDCS methodology, or even subtle priming by the experimenters (Rabipour et al., 2018, 2019). All of these variables could feasibly influence the outcome of a reflective binary probe question and these factors should now be examined systematically in further studies.

It is important to note that although the congruence of the cross‐correlations was a better classifier of participants’ sensitivity to stimulation than the end‐of‐study guess, it was only accurate in around 70% of individuals. This serves to highlight a moderate degree of inter‐individual variability in sensitivity across our 64 participants. Although the mean coefficient for congruent correlations was *r* = .53, this ranged from as low as *r* = .11 to as high as *r* = .99. We also identified sub‐groups of individuals, where some people were more susceptible to sham‐blinding than others. For example, participants who had a high (or low) sensitivity to active stimulation also tended to have a high (or low) sensitivity to sham (Figure 3, left panel), with a small correlation of *r*(64) = .26, *p* = .039 between the two protocols. It may therefore be possible to screen individuals and select those with low sensitivity during the recruitment process for research studies in order to maximise the likelihood of blinding the conditions successfully. However, this is unlikely to be sustainable when recruiting participants for clinical trials, particularly for conditions with a small patient pool, as this would result in the exclusion of a large proportion of otherwise‐eligible individuals.

It is perhaps unsurprising that we identified a greater sensitivity to high‐intensity (2 mA) tDCS compared to low‐intensity (1 mA) stimulation. At the group‐level, participants reported a clear, sustained difference between active and sham that was maintained until almost the end of the 11 min total stimulation period. In Greinacher et al. (2019), during 1 mA tDCS, this divergence lasted until 6 mins post‐onset of stimulation, with sporadic differences lasting until 11.5 mins. In fact, the current strength in the present study was a good classifier of sensitivity during the active stimulation protocol (where sensitivity was higher during 2 mA than 1 mA), but was less successful at predicting sensitivity during sham. In other words, it may be that the scalp is generally most sensitive during the first 1–2 min after stimulation onset (coinciding with the sham stimulation period), and this sensitivity is relatively independent of the strength of the current that is applied. In the active condition, the higher‐intensity current applied for a longer period results in a prolongation of the induced sensations compared to low‐intensity stimulation, thus resulting in participants being able to track the presence of stimulation more effectively throughout the duration of the experiment.

Of note, we observed a moderate positive correlation between the participants’ sensitivity and our main behavioural outcome measure. Participants who were sensitive to the presence of active 2 mA stimulation tended to slow in their reaction time at the end of the stimulation period relative to baseline. We hypothesise that these participants may have been distracted from the task by the prolonged presence of the high‐intensity stimulation on their scalp, particularly since no correlation was observed when the stimulation duration was short, during 2 mA sham, nor during the two lower‐intensity 1 mA conditions. We therefore did not find evidence of a facilitatory placebo effect in our dataset (where sensitive participants might have speeded their responses during the reaction time task), but rather the opposite. The relationship between sensitivity and behaviour remains an important question for future research.

We must acknowledge that our results, at present, only pertain to the specific experimental design, parameters and participant group tested here, and must now be replicated with different electrode sizes, montages, with older people and patient groups, and in experiments adopting a between‐group design. It could be that between‐group designs result in less pronounced differences between protocols, particularly if participants are naive to tDCS; however, repeated measures designs are commonly used in tDCS research, and are indeed preferred because of their increased statistical power. This is particularly important in a field where observed effect sizes are typically small. Secondly, as noted in Greinacher et al. (2019) we probed sham‐blinding regularly throughout the study and in doing so we may have drawn attention to the sensations more than in studies using only the traditional end‐of‐study guess questionnaire. However, in this scenario we would have expected to observe more consistently higher sensitivities across participants than we did, rather than the range (and potential subgroups) of sensitive and insensitive individuals. As we noted in Greinacher et al. (2019) we also found that reaction times during 1 mA tDCS were no different to those obtained in Minarik et al. (2016) where no online probes were used, indicating that participants were not unduly distracted from the reaction time task by the presence of the online probe questions.

Finally, these results prompt us to make a number of recommendations to improve sham blinding in future studies. Firstly, we recommend introducing at least some online probe questions during the course of stimulation to assess the effectiveness of the placebo control that is being delivered. We also recommend eliminating the traditional *fade‐in, short stimulation, fade‐out* sham protocol where possible, and adopting “active controls” instead. For example, delivering active stimulation over a cortical site that is thought not to be involved in the behaviour of interest, or alternatively by applying an anodal compared to a cathodal protocol. In some cases this approach may not be viable, for instance in patients after stroke, where it may prove detrimental to induce a further reduction (or indeed an increase) of neural activity. Topical anaesthetics should also be used where possible to minimise or eliminate sensory side‐effects (Guleyupoglu et al., 2014; McFadden et al., 2011). Recently, Neri et al. (2020) developed a novel method of sham blinding (*ActiSham*) which involves deliberately shunting the current across the scalp in a controlled manner using multifocal tDCS. This method is claimed to provide similar scalp sensations to active tDCS, without inducing a neural effect, and participants are reportedly unable to dissociate *ActiSham* from active stimulation. It would be beneficial to develop such protocols using the standard, large electrode montages which are used more widely in tDCS research. Further studies should also be carried out to quantify the time course of more specific side effects, such as headache and tingling, and whether they might predict sensitivity to stimulation in a similar manner (Fertonani et al., 2015).

## 5 CONCLUSION

High‐intensity 2 mA tDCS current results in a more prolonged and consistent period of time where the active stimulation is perceived as being switched on, compared to low‐intensity 1 mA stimulation. The accuracy of the end‐of‐study guess (i.e. the standard post‐study questionnaire method of determining whether participants can dissociate the active from the sham session) was no better than chance at predicting the sensitivity of participants to the presence of either anodal or sham tDCS. This raises important questions regarding the validity of the questionnaire‐based method of assessing sham‐blinding.

## CONFLICT OF INTEREST

The authors declare no competing interests.

## AUTHOR CONTRIBUTIONS

GL conceived and designed experiment. CT and CJ performed experiments. GL, CT, and CJ analysed the data. GL, CT, and CJ prepared the manuscript. CT and CJ contributed equally to this manuscript.

### PEER REVIEW

The peer review history for this article is available at https://publons.com/publon/10.1111/ejn.15018.

## Supporting information

Supplementary Material

## Data Availability

The pre‐registered protocol and full datasets are available at https://osf.io/4ath9/.
