# Supplementary figures and images for: Is the “end‐of‐study guess” a valid measure of sham blinding during transcranial direct current stimulation?
Source: Eur J Neurosci. 2020 Nov 20;53(5):1592–604. doi: 10.1111/ejn.15018 (PMC8048983; doi:10.1111/ejn.15018)

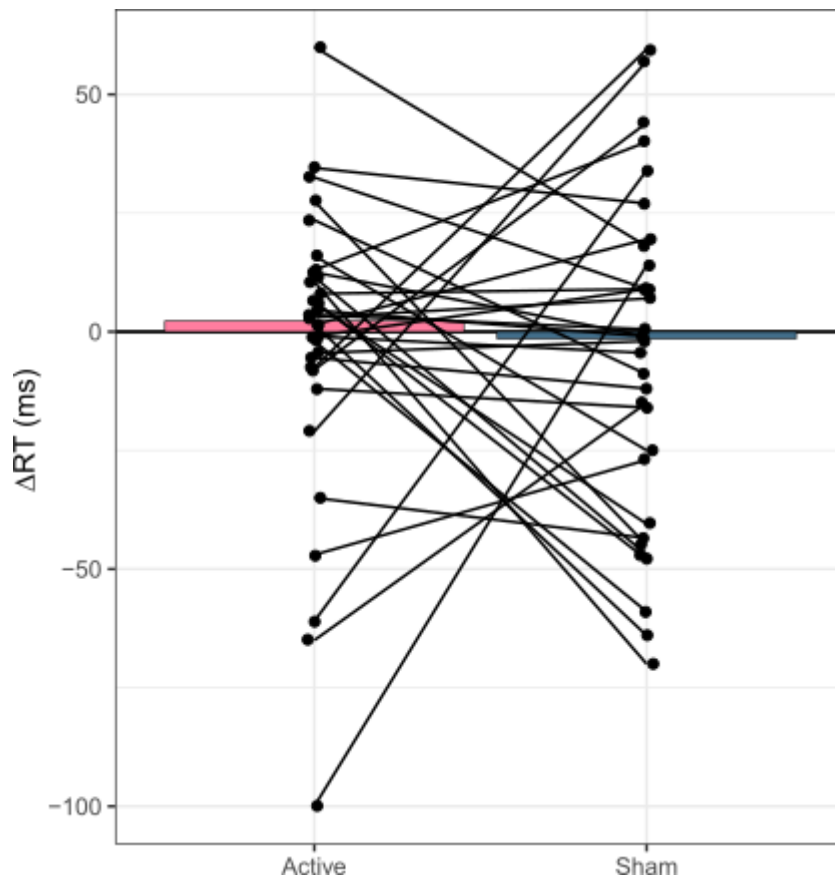

**Figure S1.** Median change in RT from baseline to Block 3 during 2mA tDCS.

Supplement: Supplementary file 1 — Supplementary Material [file EJN-53-1592-s001.pdf]
